# Empagliflozin Inhibits Basal and IL-1β-Mediated MCP-1/CCL2 and Endothelin-1 Expression in Human Proximal Tubular Cells

**DOI:** 10.3390/ijms21218189

**Published:** 2020-11-01

**Authors:** Markus Pirklbauer, Maximilian Bernd, Lisa Fuchs, Petra Staudinger, Ulrike Corazza, Johannes Leierer, Gert Mayer, Herbert Schramek

**Affiliations:** Department of Internal Medicine IV—Nephrology and Hypertension, Medical University Innsbruck, Anichstrasse 35, 6020 Innsbruck, Austria; maximilian.bernd@student.i-med.ac.at (M.B.); lisa.fuchs@i-med.ac.at (L.F.); petra.staudinger@tirol-kliniken.at (P.S.); ulrike.corazza@tirol-kliniken.at (U.C.); johannes.leierer@i-med.ac.at (J.L.); gert.mayer@i-med.ac.at (G.M.); herbert.schramek@i-med.ac.at (H.S.)

**Keywords:** SGLT2 inhibition, interleukin-1β, MCP-1/CCL2, endothelin-1, human proximal tubular cell

## Abstract

SGLT2 inhibitors (SGLT2i) slow the progression of chronic kidney disease; however, evidence for the underlying molecular mechanisms is scarce. We investigated SGLT2i-mediated effects on differential gene expression in two independent human proximal tubular cell (HPTC) lines (HK-2 and RPTEC/TERT1) at the mRNA and protein levels under normoglycemic conditions, utilizing IL-1β as a pro-inflammatory mediator. Microarray hybridization identified 259 genes that were uniformly upregulated by IL-1β (10 mg/mL) and downregulated by empagliflozin (Empa) (500 nM) after 24 h of stimulation in two independent HPTC lines (*n* = 2, each). The functional annotation of these genes identified eight pathway clusters. Among 12 genes annotated to the highest ranked cluster (enrichment score, 3.51), monocyte chemoattractant protein-1/CC-chemokine ligand 2 (MCP-1/CCL2) and endothelin-1 (ET-1) were selected for verification at mRNA and protein levels based on their established involvement in the early pathogenesis of chronic kidney disease: IL-1β upregulated basal MCP-1/CCL2 (15- and 19-fold) and ET-1 (3- and 8-fold) mRNA expression, while Empa downregulated basal MCP-1/CCL2 (0.6- and 0.5-fold) and ET-1 (0.3- and 0.2-fold) mRNA expression as early as 1 h after stimulation and for at least 24 h in HK-2 and RPTEC/TERT1 cells, respectively. The co-administration of Empa inhibited IL-1β-mediated MCP-1/CCL2 (0.2-fold, each) and ET-1 (0.2-fold, each) mRNA expression as early as 1 h after ligand stimulation and for at least 24 h in both HPTC lines, respectively. This inhibitory effect of Empa on basal and IL-1β-mediated MCP-1/CCL2 and ET-1 mRNA expression was corroborated at the protein level. Our study presents novel evidence for the interference of SGLT2 inhibition with tubular inflammatory response mechanisms under normoglycemic conditions that might account for SGLT2i-mediated nephroprotection.

## 1. Introduction

Diabetic kidney disease (DKD) is a major cause of chronic kidney disease (CKD) and end-stage renal disease worldwide [1] and markedly influences both the quality and quantity of life [2,3,4]. DKD develops in about 40% of diabetic patients [5] and increases the mortality and cardiovascular (CV) event rate further in this per se high-risk population. The management of diabetic complications consumes an enormous part of the healthcare budget in developed countries [6,7,8], and the expected 50% rise in the prevalence of diabetes mellitus within the next 30 years will further increase the associated cardio-renal disease burden [9,10,11]. From the early 1990s until recently, the inhibition of the renin–angiotensin–aldosterone system (RAAS) represented the only available treatment option to effectively slow DKD progression at least in some patients. Substantial knowledge about the complexity of DKD’s pathogenesis and multiple underlying mechanisms has been gained in recent years, allowing the investigation of emerging therapeutic strategies in large clinical trials [12]. Among type II diabetic patients, the use of anti-glycemic SGLT-2 inhibitors (SGLT2i) has been demonstrated to reduce CV risk and convey nephroprotection, both in patients at high CV risk (EMPAREG-OUTCOME, CANVAS program and DECLARE TIMI-58) [13,14,15] and with established kidney disease (CREDENCE) [16]. Recent real-world data demonstrating the nephroprotective efficiency of SGLT2i use in diabetic patients with no or mildly impaired kidney function [17,18] are further reassuring. Interestingly, SGLT2i reduce the risk of worsening heart failure or CV death independently of diabetes status [19]. Even more intriguingly, the DAPA-CKD trial recently demonstrated that dapagliflozin reduces the risk of eGFR decline, end-stage renal disease, or death from renal or CV causes in CKD patients regardless of the presence or absence of diabetes [20]. The renal benefit of SGLT2i is not associated with their HbA1c-lowering capacity but rather attributable to multiple effects that have been extensively investigated and reviewed before [12,21]: SGLT2i reduce glomerular hyperfiltration via restoring the tubuloglomerular feedback mechanism; lower body weight, plasma volume and systemic blood pressure; and reduce renal hypoxia via decreasing tubular oxygen demand. In addition, we and others previously reported that SGLT2i-mediated nephroprotection might result from direct anti-fibrotic effects at the tubular cell level. By demonstrating that SGLT2i inhibit the basal and TGF-β1-mediated expression of thrombospondin 1 (THBS1), tenascin-C (TNC) and platelet-derived growth factor-beta (PDGF-B) in two independent human proximal tubular cell (HPTC) lines under normoglycemic conditions, we recently presented non-hemodynamic, nephroprotective mechanisms of this promising class of drugs [22]. While the aforementioned mechanisms might account for the beneficial long-term effects on CKD progression, renoprotection is observed promptly after starting SGLT2i treatment. As diabetic animal studies previously reported that SGLT2i attenuates both systemic and renal tissue inflammation [23], the latter being a prominent mediator of diabetic and non-diabetic CKD [24], we aimed at elucidating the pathways involved in SGLT2i-mediated nephroprotection by using a systematic molecular approach in two independent HPTC lines, utilizing IL-1β as a pro-inflammatory mediator. By demonstrating an empagliflozin (Empa)-mediated inhibition of basal as well as IL-1β-mediated MCP-1/CCL2 and ET-1 expression under normoglycemic conditions, we present novel evidence for the interference of SGLT2 inhibition with tubular inflammatory response mechanisms that could convey SGLT2i-mediated renoprotection in normoglycemic CKD patients. 

## 2. Results

Molecular pathways that are involved in CKD pathogenesis and modifiable by SGLT2i were identified on a genome-wide level in two independent HPTC lines, namely, HK-2 and RPTEC/TERT1 cells. We specifically aimed at assessing the interference of SGLT2 inhibition with tubular inflammatory response mechanisms that are essential for the initial pathogenesis of both diabetic and non-diabetic nephropathies, by utilizing IL-1β as a pro-inflammatory ligand and Empa. Microarray hybridization analysis identified 259 genes (Table S1) that were uniformly upregulated by IL-1β (10 ng/mL) (any positive fold change, i.e., potentially involved in the tubular inflammatory response) but downregulated by Empa (500 nM) (any negative fold change, i.e., potentially involved in the suppression of tubular response mechanisms) after 24 h of stimulation in two independent HPTC lines (*n* = 2, each). Only 259 out of >30,000 genes exhibited both transcriptomic expression patterns and were subject to subsequent pathway enrichment analysis. 

**Figure 1:**
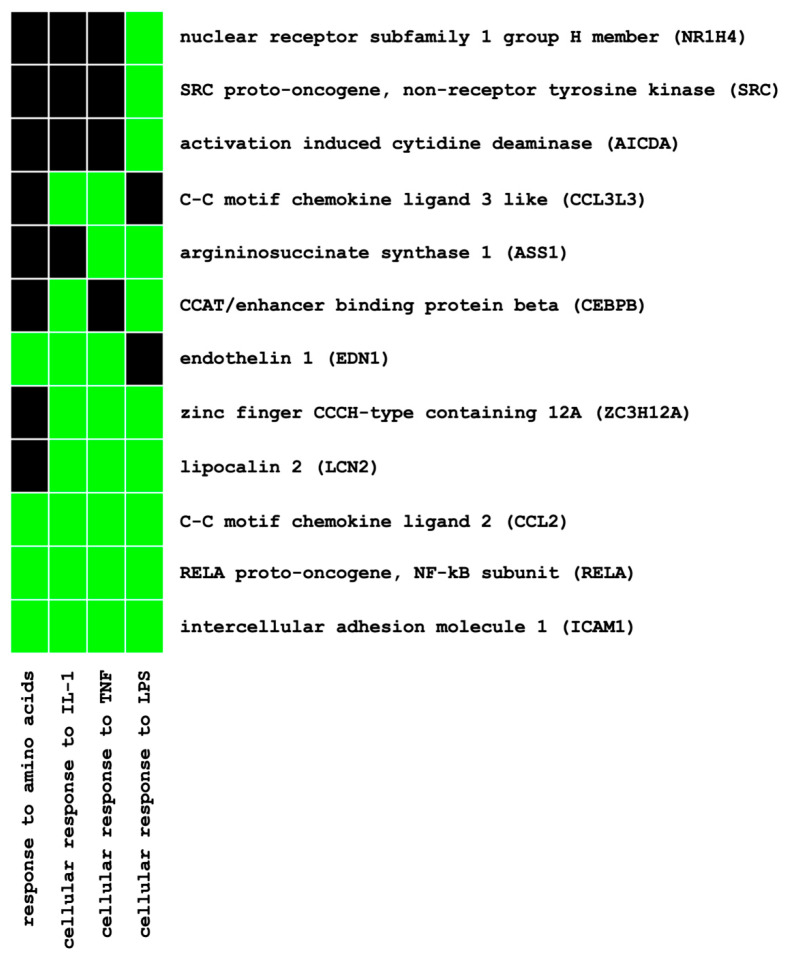
Functional annotation of differentially expressed genes using pathway enrichment analysis (DAVID): gene–pathway relationships of the highest ranked pathway cluster (enrichment score, 3.51). Established (=green) and unknown (=black) gene–pathway relationships.

The functional annotation of these genes using Database for Annotation, Visualization and Integrated Discovery (DAVID) enrichment analysis (see Materials and Methods) identified eight pathway clusters involved (EASE score < 0.05) (Table S2). Among the 12 genes annotated to the highest ranked cluster (enrichment score, 3.51) (Figure 1), two genes of interest, namely, MCP-1/CCL2 and ET-1, were selected for verification at the mRNA and protein levels based on their established involvement in early pathogenesis of diabetic and non-diabetic kidney disease.

**Figure 2:**
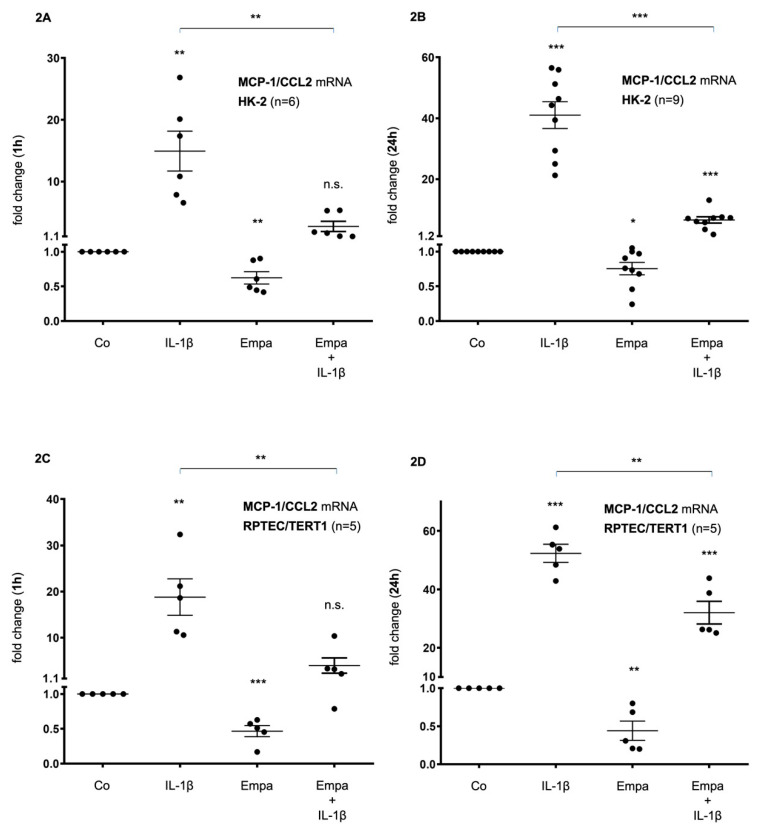
Inhibitory effect of empagliflozin on basal and IL-1β-induced MCP-1/CCL2 mRNA expression in human proximal tubular cells (HPTC). HPTCs were serum- and supplement-starved for 24 h and then stimulated for 1 h (**A**,**C**) or 24 h (**B**,**D**) with IL-1β (10 ng/mL), 500 nM empagliflozin (Empa), or a combination of Empa (500 nM) and IL-1β (10 ng/mL). Controls (Co) were left unstimulated for 1 h (**A**,**C**) or 24 h (**B**,**D**). Data are presented as fold induction above MCP-1/CCL2 control levels after normalizing to GAPDH expression. For each experimental condition, data of (n) independent experiments are presented as dots in scatter plot graphs with the respective mean ± SEM bars. (**A**,**B**) In HK-2 cells, IL-1β (10 ng/mL) led to a 15-fold and 41-fold induction of MCP-1/CCL2 mRNA expression after 1 h (**A**) and 24 h (**B**) of ligand stimulation, respectively (** *p* < 0.01 and *** *p* < 0.001, respectively, when compared with unstimulated control cells). Empa (500 nM) downregulated basal MCP-1/CCL2 mRNA expression by 0.6-fold and 0.8-fold after 1 h (**A**) and 24 h (**B**), respectively (** *p* < 0.01 and * *p* < 0.05, respectively, when compared with unstimulated control cells). Co-administration of Empa inhibited IL-1β-mediated MCP-1/CCL2 mRNA expression after 1 h (0.2-fold, ** *p* < 0.01) (**A**) and 24 h (0.16-fold, *** *p* < 0.001) (**B**). cDNA from *n* = 6 (**A**) and *n* = 9 (**B**) independent RNA isolations was used for real-time PCR analysis, respectively. (**C**,**D**) In RPTEC/TERT1 cells, IL-1β (10 ng/mL) led to a 19-fold and 52-fold induction of MCP-1/CCL2 mRNA expression after 1 h (**C**) and 24 h (**D**) of ligand stimulation, respectively (** *p* < 0.01 and *** *p* < 0.001, respectively, when compared with unstimulated control cells). Empa (500 nM) downregulated basal MCP-1/CCL2 mRNA expression by 0.5-fold and 0.44-fold after 1 h (**C**) and 24 h (**D**), respectively (*** *p* < 0.001 and ** *p* < 0.01, respectively, when compared with unstimulated control cells). Coadministration of Empa inhibited IL-1β-mediated MCP-1/CCL2 mRNA expression after 1 h (0.2-fold, ** *p* < 0.01) (**C**) and 24 h (0.6-fold, ** *p* < 0.01) (**D**). cDNAs from *n* = 5 (**C**,**D**) independent RNA isolations were used for real-time PCR analysis, each. n.s. = non-significant difference compared to control.

In HK-2 cells, basal MCP-1/CCL2 mRNA expression was upregulated by IL-1β (10 ng/mL) after 1 h (15-fold, *p* < 0.01) and 24 h (41-fold, *p* < 0.001) but downregulated by Empa (500 nM) after 1 h (0.6-fold, *p* < 0.01) and 24 h (0.8-fold, *p* < 0.05). The co-administration of Empa inhibited IL-1β-mediated MCP-1/CCL2 mRNA expression after 1 h (0.2-fold, *p* < 0.01) and 24 h (0.16-fold, *p* < 0.001) (Figure 2A,B). Concordant results were obtained in RPTEC/TERT1 cells: IL-1β (10 ng/mL) led to a 19- and 52-fold induction of basal MCP-1/CCL2 mRNA expression after 1 and 24 h (*p* < 0.01 and *p* < 0.001), respectively. Empa inhibited basal MCP-1/CCL-2 mRNA expression 0.5-fold (*p* < 0.001) and 0.44-fold (*p* < 0.01) after 1 and 24 h, respectively. The co-administration of Empa inhibited IL-1β-mediated MCP-1/CCL2 mRNA expression by 0.2-fold (*p* < 0.01) and 0.6-fold (*p* < 0.01) after 1 and 24 h, respectively (Figure 2C,D).

**Figure 3:**
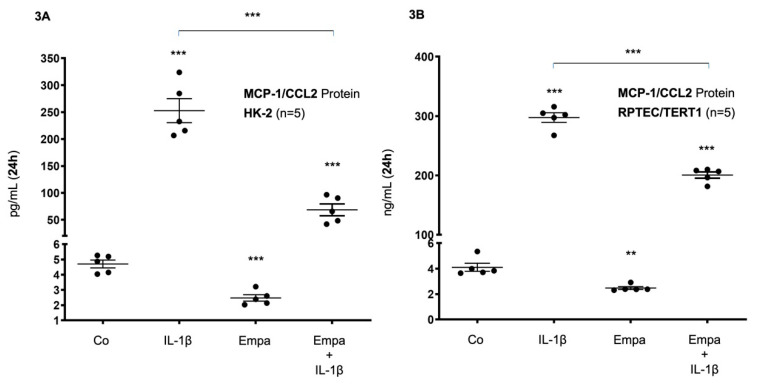
Inhibitory effect of empagliflozin on basal and IL-1β-induced MCP-1/CCL-2 protein expression in human proximal tubular cells. HPTCs were serum- and supplement-starved for 24 h and then stimulated for 24 h with IL-1β (10 ng/mL), 500 nM Empa, or a combination of Empa (500 nM) and IL-1β (10 ng/mL). Controls (Co) were left unstimulated for 24 h (**A**,**B**). Cell supernatants from *n* = 5 independent experiments were used for ELISA. Data are presented as concentrations when compared with MCP-1/CCL2 control levels. For each experimental condition, data of five independent experiments are presented as dots in scatter plot graphs with the respective mean ± SEM bars. (**A**) In HK-2 cells, IL-1β (10 ng/mL) induced MCP-1/CCL2 protein expression 54-fold (*** *p* < 0.001 compared with unstimulated control cells). Empa (500 nM) downregulated basal MCP-1/CCL2 protein expression by 0.53-fold (*** *p* < 0.001, compared with unstimulated control cells). Administration of Empa in the presence of IL-1β resulted in a 0.27-fold inhibition of IL-1β-induced MCP-1/CCL2 protein expression (*** *p* < 0.001 when compared with IL-1β-stimulated cells). (**B**) In RPTEC/TERT1 cells, IL-1β (10 ng/mL) induced MCP-1/CCL2 protein expression 73-fold (*** *p* < 0.001 compared with unstimulated control cells). Empa (500 nM) downregulated basal MCP-1/CCL2 protein expression by 0.6–fold (** *p* < 0.01, compared with unstimulated control cells). Administration of Empa in the presence of IL-1β led to a 0.67-fold inhibition of IL-1β-induced MCP-1/CCL2 protein expression (*** *p* < 0.001 when compared with IL-1β-stimulated cells).

These results were confirmed at the protein level in both HPTC lines: After 24 h of stimulation, basal MCP-1/CCL2 protein expression was upregulated by IL-1β (10 ng/mL) (54- and 73-fold, respectively, *p* < 0.001 each) and downregulated by Empa (500 nM) (0.53- and 0.6-fold, *p* < 0.001 and *p* < 0.01, respectively) in HK-2 and RPTEC/TERT1 cells, respectively (Figure 3A,B). The co-administration of Empa inhibited IL-1β-mediated MCP-1/CCL2 protein expression after 24 h (0.27- and 0.67-fold, *p* < 0.001 each) in HK-2 and RPTEC/TERT1 cells, respectively (Figure 3A,B).

**Figure 4:**
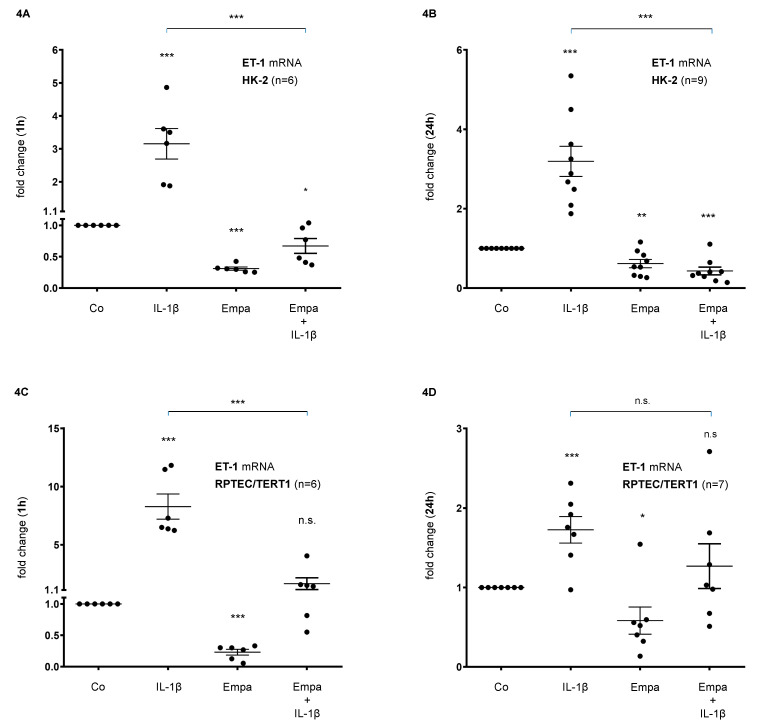
Inhibitory effect of empagliflozin on basal and IL-1β-induced ET-1 mRNA expression in human proximal tubular cells. HPTCs were serum- and supplement-starved for 24 h and then stimulated for 1 h (**A**,**B**) or 24 h (**C**,**D**) with IL-1β (10 ng/mL), 500 nM Empa, or a combination of Empa (500 nM) and IL-1β (10 ng/mL). Controls (Co) were left unstimulated for 1 h (**A**,**C**) or 24 h (**B**,**D**). Data are presented as fold induction above ET-1 control levels after normalizing to GAPDH expression. For each experimental condition, data of (n) independent experiments are presented as dots in scatter plot graphs with the respective mean ± SEM bars. (**A**,**B**) In HK-2 cells, IL-1β (10 ng/mL) stimulated ET-1 mRNA expression 3-fold (*** *p* < 0.001, each, when compared with unstimulated control cells) after 1 h (**A**) and 24 h (**B**) of ligand stimulation, respectively. Empa (500 nM) downregulated basal ET-1 mRNA expression by 0.3-fold and 0.6-fold after 1 h (**A**) and 24 h (**B**), respectively (*** *p* < 0.001 and ** *p* < 0.01, respectively, when compared with unstimulated control cells). Co-administration of Empa inhibited IL-1β-mediated ET-1 mRNA expression after 1 h (0.2-fold, *** *p* < 0.001) (**A**) and 24 h (0.1-fold, *** *p* < 0.001) (**B**). cDNA from *n* = 6 (**A**) and *n* = 9 (**B**) independent RNA isolations was used for real-time PCR analysis, respectively. (**C**,**D**) In RPTEC/TERT1 cells, IL-1β (10 ng/mL) stimulated ET-1 mRNA expression 8-fold and 1.7-fold after 1 h (**C**) and 24 h (**D**) of ligand stimulation, respectively (*** *p* < 0.001, each, when compared with unstimulated control cells). Empa (500 nM) downregulated basal ET-1 mRNA expression by 0.2-fold and 0.6-fold after 1 h (**C**) and 24 h (**D**), respectively (*** *p* < 0.001 and * *p* < 0.05, respectively, when compared with unstimulated control cells). Co-administration of Empa inhibited IL-1β-mediated ET-1 mRNA expression after 1 h (0.2-fold, *** *p* < 0.001) (***C***) and 24 h (0.7-fold, n.s.) (**D**). cDNA from *n* = 6 (**C**) and *n* = 7 (**D**) independent RNA isolations was used for real-time PCR analysis, respectively. n.s. = non-significant difference compared to control.

Basal ET-1 mRNA expression was upregulated by IL-1β (10 ng/mL) after 1 h (3-fold, *p* < 0.001) and 24 h (3-fold, *p* < 0.001) but downregulated by Empa (500 nM) 0.3-fold (*p* < 0.001) and 0.6-fold (*p* < 0.01) after 1 and 24h, respectively. The co-administration of Empa inhibited IL-1β-mediated ET-1 mRNA expression after 1 h (0.2-fold, *p* < 0.001) and 24 h (0.1-fold, *p* < 0.001) in HK-2 cells (Figure 4A,B). Accordingly, in RPTEC/TERT1 cells, IL-1β (10 ng/mL) induced basal ET-1 mRNA expression 8-fold (*p* < 0.001) and 1.7-fold (*p* < 0.001) after 1 and 24 h, respectively. Empa (500 nM) attenuated basal ET-1 mRNA expression after 1 h (0.2-fold, *p* < 0.001) and 24 h (0.6-fold, *p* < 0.05) and inhibited IL-1β-mediated ET-1 mRNA expression when co-administered with IL-1β after 1 h (0.2-fold, *p* < 0.001) and 24 h (0.7-fold, n.s.) of ligand stimulation in RPTEC/TERT1 cells (Figure 4C,D).

**Figure 5:**
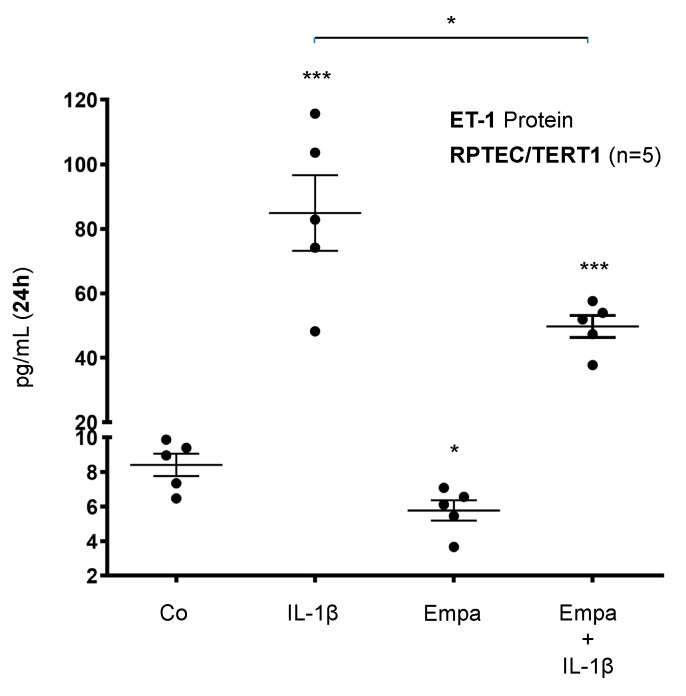
Inhibitory effect of empagliflozin on basal and IL-1β-induced ET-1 protein expression in human proximal tubular cells. RPTEC/TERT1 cells were serum- and supplement-starved for 24 h and then stimulated for 24 h with IL-1β (10 ng/mL), 500 nM Empa, or a combination of Empa (500 nM) and IL-1β (10 ng/mL). Controls (Co) were left unstimulated for 24 h. Cell supernatants from *n* = 5 independent experiments were used for ELISA. Data are presented as concentrations (pg/mL) when compared with ET-1 control levels. For each experimental condition, data of five independent experiments are presented as dots in scatter plot graphs with the respective mean ± SEM bars. IL-1β induced ET-1 protein expression 10-fold (*** *p* < 0.001 compared with unstimulated control cells). Empa downregulated basal ET-1 protein expression by 0.69-fold (* *p* < 0.05 compared with unstimulated control cells). Administration of Empa in the presence of IL-1β resulted in a 0.59-fold inhibition of IL-1β-induced ET-1 protein expression (* *p* < 0.05 when compared with IL-1β-stimulated cells).

Basal ET-1 protein expression was stimulated by IL-1β (10 ng/mL) (10-fold, *p* < 0.001) and downregulated by Empa (500 nM) (0.69-fold, *p* < 0.05) in RPTEC/TERT1 cells after 24 h of incubation. The co-administration of Empa downregulated IL-1β-induced ET-1 protein expression (0.59-fold, *p* < 0.05) in RPTEC/TERT1 cells (Figure 5).

## 3. Discussion

By demonstrating an Empa-mediated inhibition of basal and IL-1β-induced MCP-1/CCL2 as well as ET-1 expression in two independent HPTC lines under normoglycemic conditions, we present novel evidence for anti-inflammatory effects of SGLT2i that might account for SGLT2i-mediated renoprotection in CKD.

The activation of inflammatory pathways is centrally involved in the development and progression of diabetic and non-diabetic CKD [24,25], and the proximal tubule is considered one of the primary sites of damage during initial CKD pathogenesis involving tubular cell growth and the generation of reactive oxygen species, and inflammatory as well as pro-fibrotic mediators [26,27,28]. Pro-inflammatory mediators facilitate the recruitment and activation of inflammatory cells, mostly macrophages, to both the glomerular and tubulo-interstitial compartments [29,30], ultimately leading to renal damage and fibrosis. Moreover, observational studies found increased levels of circulating CRP, fibrinogen, IL-6 and TNF-alpha in patients with DKD [31,32]. Both local and systemic inflammation drives the progression of diabetic and non-diabetic kidney disease [33]. The inflammasome-mediated release of IL-1β has been demonstrated to orchestrate “sterile” inflammation and to promote the onset and progression of DKD [34]. IL-1β antagonism reduces systemic inflammation in type II diabetic patients [35] and had beneficial effects on diabetes-related CKD in murine studies [36,37]. Most recently, the inhibition of IL-1β by using a monoclonal antibody in diabetic db/db mice with progressive kidney disease was demonstrated to reduce the expression of renal damage markers and to attenuate GFR decline [38]. These findings indicate that IL-1β antagonism might be a therapeutic option in diabetic and non-diabetic CKD and emphasize the relevance of our present results showing an inhibitory effect of Empa on IL-1β-mediated gene expression in HPTCs. 

In diabetic animal studies, the use of SGLT2i reduced inflammatory markers and oxidative stress as well as glomerular and tubulo-interstitial damage [39,40,41]. Despite large-scale clinical trials showing nephroprotection promptly after initiating SGLT2i, data regarding the anti-inflammatory potential of SGLT2i in humans are rather scarce. For example, canagliflozin treatment decreased the plasma levels of biomarkers related to inflammation and fibrosis, such as tumor necrosis factor receptor 1, IL-6, matrix metalloproteinase 7 and fibronectin 1, during a two-year follow-up when analyzing samples from a clinical phase II study [42]. Dapagliflozin reduced urinary IL-6 concentrations in a post hoc analysis of a cross-over clinical trial [43]. In HK-2 cells, the high-glucose-induced expression of Toll-like receptor-4, NF-κB, collagen IV and IL-6 was attenuated by Empa [44]. At the cellular level, to date, evidence for SGLT2i-mediated anti-inflammatory potential under normoglycemic conditions is not available. To elucidate the direct effects of SGLT2i on inflammatory response mechanisms at the tubular cell level, we conducted a systematic molecular approach in two independent HPTC lines under normoglycemic conditions, utilizing IL-1β as a pro-inflammatory mediator. Potential genes of interest and related pathways were identified on a genome-wide level by using microarray analysis and the DAVID gene annotation software. Unlike previous transcriptomic studies that selected genes of interest solely based on arbitrary fold-change cut offs, we used a systematic approach by combining a genome-wide screening (i.e., the identification of distinct transcriptomic expression patterns without applying strict fold-change-based selection criteria upfront) with subsequent pathway enrichment analysis to identify potential genes of interest for further in-depth molecular analysis. Based on only 259 out of >30,000 genes (Table S1) that presented uniform expression patterns in the transcriptomic analysis of two independent HPTC lines (i.e., upregulation by IL-1β and downregulation by Empa), pathway enrichment analysis identified eight pathway clusters involved (Table S2). By focusing on the highest annotated pathway cluster (enrichment score, 3.51) and its 12 related genes (Figure 1), two genes of interest, namely, MCP-1/CCL2 and ET-1, were selected for further verification at the mRNA and protein levels based on their established involvement in the early pathogenesis of diabetic- and non-diabetic kidney disease.

The chemokine MCP-1/CCL2 is expressed by mononuclear and renal cells, facilitating the recruitment of monocytes and macrophages during inflammatory states, including renal pathologies such as lupus nephritis, IgA nephropathy, crescentic glomerulonephritis and diabetic nephropathy, via G-protein coupled CC-chemokine receptor-2 (CCR2) [45]. Diabetic animals show increased glomerular MCP-1/CCL2 expression during early disease stages [30,31], and tubular MCP-1/CCL2 overexpression has been demonstrated both in experimental [46] and human DKD [47]. Urinary MCP-1/CCL2 is increased in patients with DKD and is independently associated with rapid GFR decline [48,49]. High glucose and advanced glycation endproducts can induce tubular MCP-1 secretion in diabetic mice [46]. MCP-1/CCL2 knockout was demonstrated to inhibit the development of albuminuria and to reduce the number of atrophic tubules promptly after diabetes onset in streptozotocin-induced diabetic mice [46,50]. Strategies to experimentally interfere with the MCP-1/CCR2 system have been demonstrated to reduce glomerular hypertrophy as well as the renal expression of fibronectin, collagen type IV and the pro-fibrotic mediator TGF-ß1 [51,52]. MCP-1/CCL2 is a potential target for DKD treatment, as established nephroprotective therapies, such as RAAS inhibition, have been demonstrated to reduce renal MCP-1 expression and macrophage recruitment in experimental diabetic nephropathy [53] as well as to ameliorate albuminuria, slow GFR decline and decrease urinary MCP-1/CCL2 in type II DKD patients [54]. More recently, the inhibition of MCP-1/CCL2 using neutralizing antibodies or downstream receptor blockade has been demonstrated to reduce albuminuria and inflammation in rodent DKD models [31,55]. Emapticap pegol, an MCP-1/CCL2 inhibitor, reduced albuminuria in a phase II clinical trial [56]. Beyond its beneficial effects in DKD, the blockade of the MCP-1/CCL2 axis has demonstrated nephroprotective potential in a mouse model of non-diabetic kidney disease [45]. By demonstrating an MCP-1/CCL2-lowering effect of Empa in HPTCs, we thus present novel evidence for the anti-inflammatory potential of SGLT2i, which might have therapeutic implications beyond attenuating DKD, given the key role of inflammation for non-diabetic kidney disease as well as extra-renal diabetic complications.

The 21-amino-acid peptide ET-1 is the most abundant endothelin isoform and—as the most potent human vasoconstrictor—primarily expressed by the vascular endothelium to maintain vascular tone and blood pressure. In the kidney, ET-1 is predominantly produced by endothelial and tubular cells and has multiple auto- and paracrine effects via the activation of two G-protein coupled receptor subtypes, namely, ETA and ETB. Renal ET-1 expression is generally increased in different types of kidney disease, including DKD, and has been described to mediate vasoconstriction, proteinuria, inflammation, mesangial cell proliferation and interstitial fibrosis [57]. Selective ETA or dual ETA/ETB receptor blockade demonstrated renoprotective potential in experimental preclinical studies [58,59] as well as in recent clinical trials involving patients with diabetic and non-diabetic kidney disease [60,61,62,63]. Most recently, the SONAR trial demonstrated that atrasentan, a selective ETA receptor antagonist, reduces the risk of renal events (the doubling of serum creatinine or end-stage renal disease) in patients with diabetes and CKD (HR, 0.65; *p* < 0.005) [64]. While the renal benefit of endothelin receptor blockade seems to be consistent, the underlying protective mechanisms are far less understood but—at least partly—involve anti-inflammatory effects: the selective blockade of the ETA receptor attenuates renal macrophage infiltration as well as the urinary excretion of TGF-β and prostaglandin E2 in diabetic rats [65]. These findings are in line with previous animal studies showing that ET-1 induces renal inflammatory mechanisms, such as macrophage recruitment and cytokine release, in diabetic and non-diabetic nephropathies [66,67]. Furthermore, ETA receptor blockade maintains podocyte integrity by reducing heparinase overexpression in rodent DKD models [68,69]. To date, there are no data available regarding the pharmacologic inhibition of tubular ET-1 excretion in HPTCs. 

Our study is the first to demonstrate an SGLT2i-mediated attenuation of basal and IL-1β-induced endothlin-1 expression in two independent HPTCs at the mRNA and protein levels. Given the evidence for the involvement of ET-1 in the early (inflammatory) pathogenesis of CKD, this SGLT2i-mediated effect might—at least partially—explain the observed effectiveness of this class of drugs in large-scale clinical trials. Most interestingly, the observed anti-inflammatory effects of Empa seem to be tubular cell-specific, as ET-1 expression was not affected by canagliflozin in vascular endothelial cells and IL-1β-induced MCP-1/CCL2 and IL-6 mRNA expression was attenuated by canagliflozin but not empa- or dapagliflozin in these cells [70]. We present novel evidence for the interference of Empa with tubular inflammatory response mechanisms under normoglycemic conditions. In light of the results of the DAPA-CKD trial demonstrating SGLT2i-mediated nephroprotection in CKD patients regardless of the presence or absence of diabetes [20], our results provide evidence for one potential mechanism underlying SGLT2i-mediated renoprotection in normoglycemic CKD patients. However, further studies are necessary to elucidate the downstream molecular mechanisms of Empa-mediated effects on the tubular inflammatory response as well as to assess the potential beneficial effects of SGLT2i-mediated tubular MCP-1/CCL2 and/or ET-1 suppression in animal models and humans.

## 4. Materials and Methods 

### 4.1. Reagents

The cell culture media, including the keratinocyte growth supplement, fetal bovine serum (FBS) and GlutaMAX^TM^ supplement, were obtained from Gibco Life Technologies (Paisley, UK) via Thermo Fisher Scientific (Waltham, MA, USA). Trypsin-EDTA, penicillin–streptomycin solution, hydrocortisone, and epidermal growth factor (EGF) were purchased from Sigma Aldrich Productions GmbH (Steinheim, Germany). Insulin–Transferrin–Sodium Selenite supplement (ITS) was purchased from Roche (Basel, Switzerland). Empagliflozin (Empa) was obtained from Selleckchem (Houston, TX, USA), and recombinant human IL-1β, from R&D Systems (Minneapolis, MN, USA) via Biomedica Medizinprodukte GmbH (Wien, Austria). 

### 4.2. Cell Culture

Human kidney 2 (HK-2) cells (American Type Culture Collection, Rockville, MD, USA) as well as RPTEC/TERT1 cells (Evercyte GmBH, Vienna, Austria) were cultured under normoglycemic conditions as described previously in detail [71,72,73,74,75]. The HPTCs were serum- and supplement-starved for 24 h and used for experiments after an additional medium change. Empa (500 nM) and IL-1β (10 ng/mL) stimulation was performed in the absence of serum and growth supplements, except for ELISA-based monocyte chemoattractant protein-1/CC-chemokine ligand 2 (MCP-1/CCL2) protein measurements, where 2% fetal bovine serum (FBS) was added at the time of stimulation according to the manufacturer’s protocol. 

### 4.3. Microarray Hybridization Analysis 

Microarray hybridization analysis was conducted for each experimental condition in biological duplicates using Agilent oligonucleotide microarrays according to the Agilent One-Color Microarray protocol (Agilent Technologies, Santa Clara, CA, USA). A detailed description of the method has been published previously [22]. The microarray analysis followed the Minimum Information About a Microarray Experiment (MIAME) guidelines [76]. The complete microarray data set has been submitted to the Gene Expression Omnibus (GEO) (http://www.ncbi.nlm.nih.gov/geo/) (accession number: GSE155867).

### 4.4. Pathway Enrichment Analysis

Molecular pathway enrichment analysis using the Database for Annotation, Visualization and Integrated Discovery (DAVID) [77,78] tool was conducted for those genes that were identified to be differentially expressed (i.e., upregulated by IL-1β and downregulated by Empa) according to the conducted microarray analysis (see above). The DAVID bioinformatics database (https://david.ncifcrf.gov/) consists of an integrated biological knowledgebase and analytic data-mining tools aimed at systematically extracting biological meaning from large gene/protein lists derived from high-throughput genomic experiments (e.g., microarray analysis). The DAVID bioinformatics database allows the functional annotation of differentially expressed genes by using multiple pathway-mining tools (e.g., KEGG, Biocharta, BBID, etc.) and gene ontology terms, thereby enabling an in-depth understanding of the biological themes that are enriched in HPTCs. The Expression Analysis Systematic Explorer (EASE) score, a statistical test for the over-representation of annotation classes, was computed. Annotation categories with an EASE score of less than 0.05 were considered statistically significant.

### 4.5. Ribonucleic Acid (RNA) Extraction and Real-Time PCR Analysis

Total RNA was isolated from HPTCs according to the manufacturer’s protocol for the innuPREP RNA Mini Kit 2.0 (Analytik Jena AG, Jena, Germany). The RNA yield was determined using a spectrophotometer (DeNovix, Wilmington, DE, USA). cDNA was generated by the reverse transcription of RNA using the High Capacity cDNA Reverse Transcription kit (Applied Biosystems, Foster City, CA, USA) and then analyzed on a 7500 Fast Real-Time PCR System (Applied Biosystems, Foster City, CA, USA) utilizing the respective TaqMan^®^ Gene Expression Assays (Gibco Life Technologies, Paisley, UK, via Thermo Fisher Scientific, Waltham, MA, USA): MCP-1/CCL2 (Hs00234140_m1), endothelin-1 (ET-1) (Hs00174961_m1) and GAPDH (Hs99999905_m1). The 2^−ΔΔCT^ method was used for the relative quantification of the real-time PCR data, utilizing GAPDH as a reference gene. A detailed description of the methods used has been given elsewhere [22].

### 4.6. Enzyme-Linked Immunosorbent Assays

MCP-1/CCL2 and ET-1 protein expression was measured in cell supernatants. The analysis of protein expression was performed using commercially available enzyme-linked immunosorbent assay (ELISA) kits according to the manufacturer’s instructions. The following ELISA kits were purchased: human MCP-1/CCL2 (ELH-MCP1) (RayBiotech Life, Peachtree Corners, GA, USA) and (DCP00) (R&D Systems, Minneapolis, MN, USA), and human ET-1 (DET100) (R&D Systems, Minneapolis, MN, USA).

### 4.7. Cell Viability

Cell viability was assessed for each experimental condition by using an MTT (3-(4,5-dimethylthiazol-2-yl)-2,5-diphenyltetrazolium bromide) assay (Cell Proliferation Kit I, Roche Diagnostics GmbH, Penzberg, Germany) according to the manufacturer’s instructions. The method has been described in detail previously [22]. The cell viability did not significantly differ between the experimental conditions.

### 4.8. Statistical Analyses

The pathway enrichment analysis was statistically evaluated by calculating the *Expression Analysis Systematic Explorer* (EASE) score. An EASE score < 0.05 reflects a statistically significant over-representation of annotation classes. The data of the quantitative real-time PCR and ELISA are presented as dots in the scatter plot graphs and as mean values ± SEM bars for each experimental condition. Groups were compared by unpaired t-tests, and a *p*-value < 0.05 was considered statistically significant.

## Supplementary Materials

Supplementary materials can be found at https://www.mdpi.com/1422-0067/21/21/8189/s1. Table S1: Genome-wide identification of SGLT2i’s interaction with early inflammatory response in human proximal tubular cells. Table S2: Pathway annotation clustering based on 259 genes that show a distinct genome-wide expression pattern (i.e., IL-1β-meditated upregulation and Empa-mediated downregulation) in two independent human proximal tubular cell lines.

## Abbreviations

CCR2	CC-chemokine receptor-2
cDNA	Complementary deoxyribonucleic acid
CKD	Chronic kidney disease
CV	Cardiovascular
CT	Cycle threshold
DAVID	Database for Annotation, Visualization and Integrated Discovery
DKD	Diabetic kidney disease
EASE	Expression Analysis Systematic Explorer
EGF	Epidermal growth factor
ELISA	Enzyme-linked immunosorbent assay
Empa	Empagliflozin
ET-1	Endothelin-1
FBS	Fetal bovine serum
GAPDH	Glyceraldehyde-3-phosphate dehydrogenase
HK-2	Human kidney-2
HPTC	Human proximal tubular cell
IL-1ß	Interleukin-1ß
ITS	Insulin–Transferrin–Sodium Selenite supplement
MCP-1/CCL2	Monocyte chemoattractant protein-1/CC-chemokine ligand 2
MIAME	Minimum Information About a Microarray Experiment
mRNA	Messenger ribonucleic acid
MTT	3-(4,5-dimethylthiazol-2-yl)-2,5-diphenyltetrazolium bromide
PCR	Polymerase chain reaction
PDGF-B	Platelet-derived growth factor-ß
RAAS	Renin–angiotensin–aldosterone system
RNA	Ribonucleic acid
RPTEC/TERT1	Renal proximal tubule epithelial cell/TERT-immortalized 1
SEM	Standard error of the mean
SGLT2	Sodium–glucose co-transporter 2
SGLT2i	SGLT2 inhibitors
THBS1	Thrombospondin-1
TNC	Tenascin-C
