# Supplementary material for: Empagliflozin Inhibits Basal and IL-1β-Mediated MCP-1/CCL2 and Endothelin-1 Expression in Human Proximal Tubular Cells
Source: Int J Mol Sci. 2020 Nov 1;21(21):8189. doi: 10.3390/ijms21218189 (PMC7663377; doi:10.3390/ijms21218189)
Supplement: Supplementary file 1 [file ijms-21-08189-s001.pdf]

Table S1: Genome-wide identification of SGLT2i`s interaction with early inflammatory response in human proximal tubular cells.

Systematic Name	Gene Name	Systematic Name	Gene Name
NM_001710	complement factor B(CFB)	NM_052831	solute carrier family 18 member B1(SLC18B1)
NM_031372	heterogeneous nuclear ribonucleoprotein D like(HNRNPDL)	NM_170711	DAZ associated protein 1(DAZAP1)
NM_014299	bromodomain containing 4(BRD4)	NM_001261	cyclin dependent kinase 9(CDK9)
NM_182628	cilia and flagella associated protein 100(CFAP100)	NM_178835	zinc finger protein 827(ZNF827)
NM_017906	PAK1 interacting protein 1(PAK1IP1)	NM_024015	homeobox B4(HOXB4)
NM_053279	family with sequence similarity 167 member A(FAM167A)	NM_015114	ankyrin repeat and LEM domain containing 2(ANKLE2)
NM_001031628	small cell adhesion glycoprotein(SMAGP)	NM_005721	ARP3 actin related protein 3 homolog(ACTR3)
NM_147686	TRAF3 interacting protein 2(TRAF3IP2)	NM_005720	actin related protein 2/3 complex subunit 1B(ARPC1B)
NM_018664	basic leucine zipper ATF-like transcription factor 3(BATF3)	NM_182898	cAMP responsive element binding protein 5(CREB5)
NM_025079	zinc finger CCCH-type containing 12A(ZC3H12A)	NM_020661	activation induced cytidine deaminase(AICDA)
NM_001511	C-X-C motif chemokine ligand 1(CXCL1)	NM_015213	DENN domain containing 5A(DENND5A)
NM_025072	prostaglandin E synthase 2(PTGES2)	NM_004665	vanin 2(VNN2)
NM_001024465	superoxide dismutase 2, mitochondrial(SOD2)	NM_016070	mitochondrial ribosomal protein S23(MRPS23)
NM_033199	urocortin 2(UCN2)	NM_004973	jumonji and AT-rich interaction domain containing 2(JARID2)
NM_000610	CD44 molecule (Indian blood group)(CD44)	NM_138575	PGAM family member 5, mitochondrial serine/threonine protein phosphatase(PGAM5)
NM_001077594	exocyst complex component 3 like 4(EXOC3L4)	NM_003029	SHC adaptor protein 1(SHC1)
NM_014945	actin binding LIM protein family member 3(ABLIM3)	NM_001856	collagen type XVI alpha 1 chain(COL16A1)
NM_003341	ubiquitin conjugating enzyme E2 E1(UBE2E1)	NM_198404	potassium channel tetramerization domain containing 4(KCTD4)
NM_000617	solute carrier family 11 member 2(SLC11A2)	NM_004574	septin 4(SEPT4)
NM_004901	ectonucleoside triphosphate diphosphohydrolase 4(ENTPD4)	NM_002514	nephroblastoma overexpressed(NOV)
NM_003442	zinc finger protein 143(ZNF143)	NM_001008895	cullin 4A(CUL4A)
NM_003234	transferrin receptor(TFRC)	NM_004599	sterol regulatory element binding transcription factor 2(SREBF2)
NM_002662	phospholipase D1(PLD1)	NM_006503	proteasome 26S subunit, ATPase 4(PSMC4)
NM_139239	NFKB inhibitor delta(NFKBID)	NM_001278081	uncharacterized LOC388282(LOC388282)
NM_006331	EMG1, N1-specific pseudouridine methyltransferase(EMG1)	NM_001039182	bolA family member 2B(BOLA2B)
NM_130439	MAX interactor 1, dimerization protein(MXI1)	NR_002776	MCM3AP antisense RNA 1(MCM3AP-AS1)
NM_000997	ribosomal protein L37(RPL37)	NM_005178	B-cell CLL/lymphoma 3(BCL3)
NM_001079807	pepsinogen 3, group I (pepsinogen A)(PGA3)	NM_004458	acyl-CoA synthetase long-chain family member 4(ACSL4)
NM_001017402	laminin subunit beta 3(LAMB3)	NM_001278513	cullin 4A(CUL4A)

NM_194296	spermatogenesis associated 24(SPATA24)	NM_000050	argininosuccinate synthase 1(ASS1)
NM_001001437	C-C motif chemokine ligand 3 like 3(CCL3L3)	NM_001888	crystallin mu(CRYM)
NM_001039707	serologically defined colon cancer antigen 3(SDCCAG3)	NM_000432	myosin light chain 2(MYL2)
NM_152899	interleukin 4 induced 1(IL4I1)	NM_004846	eukaryotic translation initiation factor 4E family member 2(EIF4E2)
NM_018371	chondroitin sulfate N-acetylgalactosaminyltransferase 1(CSGALNACT1)	NM_015307	family with sequence similarity 189 member A1(FAM189A1)
NM_030912	tripartite motif containing 8(TRIM8)	NM_033247	promyelocytic leukemia(PML)
NM_031453	family with sequence similarity 107 member B(FAM107B)	NM_203403	leucine rich adaptor protein 1 like(LURAP1L)
NM_030915	limb bud and heart development(LBH)	NM_000836	glutamate ionotropic receptor NMDA type subunit 2D(GRIN2D)
NR_024363	family with sequence similarity 86, member A pseudogene(FAM86B3P)	NM_006291	TNF alpha induced protein 2(TNFAIP2)
NM_003092	small nuclear ribonucleoprotein polypeptide B2(SNRPB2)	NM_015703	ribosomal RNA processing 7 homolog A(RRP7A)
NM_201434	RAB5C, member RAS oncogene family(RAB5C)	NM_015704	desumoylating isopeptidase 1(DESI1)
NM_000962	prostaglandin-endoperoxide synthase 1(PTGS1)	NM_198179	pyroglutamylated RFamide peptide receptor(QRFPR)
NM_001042414	paraspeckle component 1(PSPC1)	NM_004995	matrix metallopeptidase 14(MMP14)
NM_006494	ETS2 repressor factor(ERF)	NM_004994	matrix metallopeptidase 9(MMP9)
NM_024334	transmembrane protein 43(TMEM43)	NM_004451	estrogen related receptor alpha(ESRRA)
NM_006988	ADAM metallopeptidase with thrombospondin type 1 motif 1(ADAMTS1)	NM_020133	1-acylglycerol-3-phosphate O-acyltransferase 4(AGPAT4)
NM_004240	thyroid hormone receptor interactor 10(TRIP10)	NM_152713	STT3A, catalytic subunit of the oligosaccharyltransferase complex(STT3A)
NM_021975	RELA proto-oncogene, NF-kB subunit(RELA)	NM_022481	ArfGAP with RhoGAP domain, ankyrin repeat and PH domain 3(ARAP3)
NM_006305	acidic nuclear phosphoprotein 32 family member A(ANP32A)	NM_000064	complement C3(C3)
NM_001098213	histamine receptor H1(HRH1)	NM_005564	lipocalin 2(LCN2)
NM_006303	aminoacyl tRNA synthetase complex interacting multifunctional protein 2(AIMP2)	NM_001975	enolase 2(ENO2)
NM_002089	C-X-C motif chemokine ligand 2(CXCL2)	NM_004126	G protein subunit gamma 11(GNG11)
NM_001031855	LON peptidase N-terminal domain and ring finger 3(LONRF3)	NM_001280790	surfeit 4(SURF4)
NM_016391	NOP16 nucleolar protein(NOP16)	NM_004128	general transcription factor IIF subunit 2(GTF2F2)
NM_000777	cytochrome P450 family 3 subfamily A member 5(CYP3A5)	NM_175834	keratin 79(KRT79)
NM_182639	HPS1, biogenesis of lysosomal organelles complex 3 subunit 1(HPS1)	NM_152406	actin filament associated protein 1 like 1(AFAP1L1)
NM_032227	transmembrane protein 164(TMEM164)	NM_198951	transglutaminase 2(TGM2)
NM_005475	SH2B adaptor protein 3(SH2B3)	NM_032977	caspase 10(CASP10)
NM_014698	transmembrane protein 63A(TMEM63A)	NM_016594	FK506 binding protein 11(FKBP11)

NM_002982	C-C motif chemokine ligand 2(CCL2)	NM_181847	adhesion molecule with Ig like domain 2(AMIGO2)
NM_021095	solute carrier family 5 member 6(SLC5A6)	NM_021249	sorting nexin 6(SNX6)
NM_004730	eukaryotic translation termination factor 1(ETF1)	NM_181425	frataxin(FXN)
NM_003879	CASP8 and FADD like apoptosis regulator(CFLAR)	NM_016613	family with sequence similarity 198 member B(FAM198B)
NM_001114937	SH2 domain containing 1A(SH2D1A)	NM_001282695	family with sequence similarity 107 member B(FAM107B)
NM_001753	caveolin 1(CAV1)	NM_005253	FOS like 2, AP-1 transcription factor subunit(FOSL2)
NM_020962	immunoglobulin superfamily DCC subclass member 4(IGDCC4)	NM_014260	prefoldin subunit 6(PFDN6)
NM_000641	interleukin 11(IL11)	NM_020639	receptor interacting serine/threonine kinase 4(RIPK4)
NM_002638	peptidase inhibitor 3(PI3)	NM_001039574	potassium voltage-gated channel subfamily C member 4(KCNC4)
NM_173076	ATP binding cassette subfamily A member 12(ABCA12)	NM_172212	colony stimulating factor 1(CSF1)
NM_006244	protein phosphatase 2 regulatory subunit B'beta(PPP2R5B)	NM_007283	monoglyceride lipase(MGLL)
NM_001781	CD69 molecule(CD69)	NM_004442	EPH receptor B2(EPHB2)
NM_024102	WD repeat domain 77(WDR77)	NM_080927	discoidin, CUB and LCCL domain containing 2(DCBLD2)
NM_001288758	ras homolog family member T1(RHOT1)	NM_004441	EPH receptor B1(EPHB1)
NM_005194	CCAAT/enhancer binding protein beta(CEBPB)	NM_002160	tenascin C(TNC)
NM_005755	Epstein-Barr virus induced 3(EBI3)	NM_014395	dual adaptor of phosphotyrosine and 3-phosphoinositides 1(DAPP1)
NM_182981	oxidative stress induced growth inhibitor 1(OSGIN1)	NM_178507	out at first homolog(OAF)
NM_144573	nexilin F-actin binding protein(NEXN)	NM_003687	PDZ and LIM domain 4(PDLIM4)
NM_016037	UTP11, small subunit processome component homolog (S. cerevisiae)(UTP11)	NM_174983	major facilitator superfamily domain containing 12(MFSD12)
NM_024316	leukocyte receptor cluster member 1(LENG1)	NM_012121	CDC42 effector protein 4(CDC42EP4)
NM_001628	aldo-keto reductase family 1 member B(AKR1B1)	NM_002460	interferon regulatory factor 4(IRF4)
NM_133510	RAD51 paralog B(RAD51B)	NM_001001791	chromosome 10 open reading frame 55(C10orf55)
NM_013352	dermatan sulfate epimerase(DSE)	NM_030927	tetraspanin 14(TSPAN14)
NM_016371	hydroxysteroid 17-beta dehydrogenase 7(HSD17B7)	NM_003811	tumor necrosis factor superfamily member 9(TNFSF9)
NM_024112	chromosome 9 open reading frame 16(C9orf16)	NM_003810	tumor necrosis factor superfamily member 10(TNFSF10)
NM_177963	synaptotagmin 12(SYT12)	NM_001029	ribosomal protein S26(RPS26)
NM_001291983	protein tyrosine phosphatase, receptor type K(PTPRK)	NM_024065	phosducin like 3(PDCL3)
NM_005764	PDZK1 interacting protein 1(PDZK1IP1)	NM_005438	FOS like 1, AP-1 transcription factor subunit(FOSL1)
NM_006065	signal regulatory protein beta 1(SIRPB1)	NM_032181	eva-1 homolog A, regulator of programmed cell death(EVA1A)
NM_003064	secretory leukocyte peptidase inhibitor(SLPI)	NM_001201329	protein phosphatase 1 regulatory subunit 3B(PPP1R3B)

NM_014800	engulfment and cell motility 1(ELMO1)	NM_014388	digestive organ expansion factor homolog (zebrafish)(DIEXF)
NM_173651	fibrous sheath interacting protein 2(FSIP2)	NM_001018116	muscle related coiled-coil protein(MURC)
NM_004120	guanylate binding protein 2(GBP2)	NM_001955	endothelin 1(EDN1)
NM_144569	SPOC domain containing 1(SPOCD1)	NM_001425	epithelial membrane protein 3(EMP3)
NM_145206	vesicle transport through interaction with t-SNAREs 1A(VTI1A)	NM_005627	serum/glucocorticoid regulated kinase 1(SGK1)
NM_032375	AKT1 substrate 1(AKT1S1)	NM_001144070	ATP binding cassette subfamily C member 3(ABCC3)
NM_004613	transglutaminase 2(TGM2)	NM_012323	MAF bZIP transcription factor F(MAFF)
NM_000201	intercellular adhesion molecule 1(ICAM1)	NM_033292	caspase 1(CASP1)
NM_004494	hepatoma-derived growth factor(HDGF)	NM_017872	tRNA-histidine guanylyltransferase 1 like(THG1L)
NM_002230	junction plakoglobin(JUP)	NM_001146702	lysine demethylase 5C(KDM5C)
NM_001105558	WEE1 homolog 2(WEE2)	NM_002960	S100 calcium binding protein A3(S100A3)
NM_001040058	secreted phosphoprotein 1(SPP1)	NM_004419	dual specificity phosphatase 5(DUSP5)
NM_006764	interferon related developmental regulator 2(IFRD2)	NM_000496	crystallin beta B2(CRYBB2)
NM_003370	vasodilator-stimulated phosphoprotein(VASP)	NM_002569	furin, paired basic amino acid cleaving enzyme(FURIN)
NM_006762	lysosomal protein transmembrane 5(LAPTM5)	NM_005426	tumor protein p53 binding protein 2(TP53BP2)
NM_001731	BTG anti-proliferation factor 1(BTG1)	NM_012129	claudin 12(CLDN12)
NM_001164431	Rho GTPase activating protein 40(ARHGAP40)	NM_017491	WD repeat domain 1(WDR1)
NM_001234	caveolin 3(CAV3)	NM_000094	collagen type VII alpha 1 chain(COL7A1)
NM_006033	lipase G, endothelial type(LIPG)	NM_031959	keratin associated protein 3-2(KRTAP3-2)
NM_178181	CUB domain containing protein 1(CDCP1)	NM_005630	solute carrier organic anion transporter family member 2A1(SLCO2A1)
NM_021830	twinkle mtDNA helicase(TWNK)	NM_005429	vascular endothelial growth factor C(VEGFC)
NM_177439	FtsJ RNA methyltransferase homolog 1 (E. coli)(FTSJ1)	NM_020894	UV stimulated scaffold protein A(UVSSA)
NM_002644	polymeric immunoglobulin receptor(PIGR)	NM_002421	matrix metallopeptidase 1(MMP1)
NM_016525	ubiquitin associated protein 1(UBAP1)	NM_004073	polo like kinase 3(PLK3)
NM_152862	actin related protein 2/3 complex subunit 2(ARPC2)	NM_005123	nuclear receptor subfamily 1 group H member 4(NR1H4)
NM_016354	solute carrier organic anion transporter family member 4A1(SLCO4A1)	NM_001252406	zinc finger and BTB domain containing 7B(ZBTB7B)
NM_016931	NADPH oxidase 4(NOX4)	NM_002970	spermidine/spermine N1-acetyltransferase 1(SAT1)
NM_170685	tachykinin 4 (hemokinin)(TAC4)	NM_182920	ADAM metallopeptidase with thrombospondin type 1 motif 9(ADAMTS9)
NM_000877	interleukin 1 receptor type 1(IL1R1)	NM_014467	sushi repeat containing protein, X-linked 2(SRPX2)
NM_001195278	transmembrane protein 178B(TMEM178B)	NM_198582	kelch like family member 30(KLHL30)

NM_006528	tissue factor pathway inhibitor 2(TFPI2)	NM_138689	protein phosphatase 1 regulatory inhibitor subunit 14B(PPP1R14B)
NM_002306	galectin 3(LGALS3)	NM_013993	discoidin domain receptor tyrosine kinase 1(DDR1)
NM_002014	FK506 binding protein 4(FKBP4)	NM_203380	acyl-CoA synthetase long-chain family member 5(ACSL5)
NM_024297	PHD finger protein 23(PHF23)	NM_001144978	methylenetetrahydrofolate dehydrogenase (NADP+ dependent) 2-like(MTHFD2L)
NM_002309	leukemia inhibitory factor(LIF)	NM_138966	neuropilin and tolloid like 1(NETO1)
NM_023013	PRAME family member 1(PRAMEF1)	NM_152553	ring finger protein 217(RNF217)
NM_015714	G0/G1 switch 2(G0S2)	NM_018340	calcineurin like phosphoesterase domain containing 1(CPPED1)
NM_016084	ras related dexamethasone induced 1(RASD1)	NM_001285486	neuralized E3 ubiquitin protein ligase 3(NEURL3)
NM_001302946	tRNA nucleotidyl transferase 1(TRNT1)	NM_005417	SRC proto-oncogene, non-receptor tyrosine kinase(SRC)
NM_014359	opticin(OPTC)	NM_005418	suppression of tumorigenicity 5(ST5)
NM_006936	small ubiquitin-like modifier 3(SUMO3)	NM_004278	phosphatidylinositol glycan anchor biosynthesis class L(PIGL)

Legend Table 1: Microarray hybridization analysis identified 259 genes that were upregulated by IL-1β (any positive fold change) and downregulated by Empagliflocin (any negative fold change) in two independent human proximal tubular cell lines (HK-2 and RPTEC/TERT1) and two independent experiments (n = 2).

Table S2: Pathway annotation clustering based on 259 genes that show a distinct genome-wide expression pattern (i.e. IL-1β-meditated upregulation and Empa-mediated downregulation) in two independent human proximal tubular cell lines.

**Annotation Cluster 1**	**Enrichment Score: 3.51**	
**Database**	**Term**	**p-value**
GOTERM_BP_DIRECT	cellular response to lipopolysaccharide	2.5×10^{-5}
GOTERM_BP_DIRECT	cellular response to interleukin-1	5.2×10^{-5}
GOTERM_BP_DIRECT	cellular response to tumor necrosis factor	8.0×10^{-4}
GOTERM_BP_DIRECT	response to amino acid	8.6×10^{-3}
**Annotation Cluster 2**	**Enrichment Score: 2.68**	
**Database**	**Term**	**p-value**
SMART	BRLZ	7.1×10^{-4}
INTERPRO	Basic-leucine zipper domain	7.2×10^{-4}
UP_SEQ_FEATURE	domain:Leucine-zipper	3.0×10^{-3}
UP_SEQ_FEATURE	DNA-binding region:Basic motif	4.5×10^{-3}
INTERPRO	Fos transforming protein	5.9×10^{-3}
**Annotation Cluster 3**	**Enrichment Score: 2.34**	
**Database**	**Term**	**p-value**
GOTERM_CC_DIRECT	extracellular space	6.6×10^{-5}
UP_KEYWORDS	Secreted	3.2×10^{-3}
GOTERM_CC_DIRECT	extracellular region	1.8×10^{-2}
UP_SEQ_FEATURE	signal peptide	1.8×10^{-2}
UP_KEYWORDS	Disulfide bond	2.9×10^{-2}
**Annotation Cluster 4**	**Enrichment Score: 1.99**	
**Database**	**Term**	**p-value**
UP_KEYWORDS	Cytokine	1.2×10^{-4}
KEGG_PATHWAY	Salmonella infection	3.6×10^{-3}
GOTERM_MF_DIRECT	cytokine activity	1.1×10^{-2}
GOTERM_BP_DIRECT	cell chemotaxis	1.2×10^{-2}
KEGG_PATHWAY	Legionellosis	1.5×10^{-2}
KEGG_PATHWAY	NOD-like receptor signaling pathway	1.7×10^{-2}
KEGG_PATHWAY	Chemokine signaling pathway	1.8×10^{-2}
SMART	SCY	2.2×10^{-2}
INTERPRO	Chemokine interleukin-8-like domain	2.3×10^{-2}
KEGG_PATHWAY	Cytokine-cytokine receptor interaction	3.0×10^{-2}
GOTERM_MF_DIRECT	chemokine activity	3.0×10^{-2}
**Annotation Cluster 5**	**Enrichment Score: 1.98**	
**Database**	**Term**	**p-value**
GOTERM_BP_DIRECT	ephrin receptor signaling pathway	1.2×10^{-3}
KEGG_PATHWAY	Shigellosis	5.5×10^{-3}

GOTERM_CC_DIRECT	Arp2/3 protein complex	1.0×10^{-2}
BIOCARTA	Rho cell motility signaling pathway	1.9×10^{-2}
GOTERM_BP_DIRECT	movement of cell or subcellular component	3.0×10^{-2}
GOTERM_BP_DIRECT	Arp2/3 complex-mediated actin nucleation	3.6×10^{-2}
**Annotation Cluster 6**	**Enrichment Score: 1.86**	
**Database**	**Term**	**p-value**
UP_SEQ_FEATURE	domain:Fibronectin type-III 2	6.6×10^{-3}
UP_SEQ_FEATURE	domain:Fibronectin type-III 1	6.9×10^{-3}
SMART	FN3	1.9×10^{-2}
UP_SEQ_FEATURE	domain:Fibronectin type-III 4	4.4×10^{-2}
**Annotation Cluster 7**	**Enrichment Score: 1.78**	
**Database**	**Term**	**p-value**
GOTERM_BP_DIRECT	extracellular matrix disassembly	6.1×10^{-4}
UP_KEYWORDS	Zymogen	1.3×10^{-3}
UP_SEQ_FEATURE	short sequence motif:Cysteine switch	2.4×10^{-3}
GOTERM_BP_DIRECT	endodermal cell differentiation	5.8×10^{-3}
GOTERM_BP_DIRECT	collagen catabolic process	1.1×10^{-2}
UP_KEYWORDS	Extracellular matrix	1.4×10^{-2}
INTERPRO	Peptidase M10A, cysteine switch, zinc binding site	1.6×10^{-2}
GOTERM_BP_DIRECT	proteolysis	2.1×10^{-2}
INTERPRO	Hemopexin, conserved site	2.1×10^{-2}
INTERPRO	Metallopeptidase, catalytic domain	2.2×10^{-2}
GOTERM_CC_DIRECT	proteinaceous extracellular matrix	2.4×10^{-2}
INTERPRO	Peptidoglycan binding-like	2.8×10^{-2}
UP_SEQ_FEATURE	domain:Hemopexin-like 3	3.1×10^{-2}
UP_SEQ_FEATURE	domain:Hemopexin-like 4	3.1×10^{-2}
UP_SEQ_FEATURE	domain:Hemopexin-like 1	3.3×10^{-2}
UP_SEQ_FEATURE	domain:Hemopexin-like 2	3.3×10^{-2}
INTERPRO	Hemopexin-like repeats	3.7×10^{-2}
INTERPRO	Peptidase M10A	3.7×10^{-2}
INTERPRO	Hemopexin-like domain	3.7×10^{-2}
INTERPRO	Peptidase M10, metallopeptidase	3.7×10^{-2}
GOTERM_MF_DIRECT	endopeptidase activity	3.8×10^{-2}
SMART	HX	4.1×10^{-2}
**Annotation Cluster 8**	**Enrichment Score: 1.76**	
**Database**	**Term**	**p-value**
SMART	CASc	1.4×10^{-2}
INTERPRO	Peptidase C14, caspase precursor p45, core	1.4×10^{-2}
INTERPRO	Peptidase C14, ICE, catalytic subunit p20	1.6×10^{-2}
GOTERM_BP_DIRECT	regulation of apoptotic process	2.8×10^{-2}
